# Effectiveness of the First and Second Severe Acute Respiratory Syndrome Coronavirus 2 Vaccine Dose: A Nationwide Cohort Study From Austria on Hybrid Versus Natural Immunity

**DOI:** 10.1093/ofid/ofae547

**Published:** 2024-09-19

**Authors:** Alena Chalupka, Uwe Riedmann, Lukas Richter, Ali Chakeri, Ziad El-Khatib, Martin Sprenger, Verena Theiler-Schwetz, Christian Trummer, Peter Willeit, Harald Schennach, Bernhard Benka, Dirk Werber, Tracy Beth Høeg, John P A Ioannidis, Stefan Pilz

**Affiliations:** Division of Endocrinology and Diabetology, Department of Internal Medicine, Medical University of Graz, Graz, Austria; Institute for Surveillance and Infectious Disease Epidemiology, Austrian Agency for Health and Food Safety, Vienna, Austria; Division of Endocrinology and Diabetology, Department of Internal Medicine, Medical University of Graz, Graz, Austria; Institute for Surveillance and Infectious Disease Epidemiology, Austrian Agency for Health and Food Safety, Vienna, Austria; Institute of Statistics, Graz University of Technology, Graz, Austria; Institute for Surveillance and Infectious Disease Epidemiology, Austrian Agency for Health and Food Safety, Vienna, Austria; Centre for Public Health, Medical University Vienna, Vienna, Austria; Institute for Surveillance and Infectious Disease Epidemiology, Austrian Agency for Health and Food Safety, Vienna, Austria; Institute of Social Medicine and Epidemiology, Medical University Graz, Graz, Austria; Division of Endocrinology and Diabetology, Department of Internal Medicine, Medical University of Graz, Graz, Austria; Division of Endocrinology and Diabetology, Department of Internal Medicine, Medical University of Graz, Graz, Austria; Institute of Clinical Epidemiology, Public Health, Health Economics, Medical Statistics and Informatics, Medical University of Innsbruck, Innsbruck, Austria; Department of Public Health and Primary Care, University of Cambridge, Cambridge, United Kingdom; Ignaz Semmelweis Institute, Interuniversity Institute for Infection Research, Vienna, Austria; Central Institute for Blood Transfusion and Department of Immunology, Tirol Kliniken GmbH, Innsbruck, Austria; Institute for Surveillance and Infectious Disease Epidemiology, Austrian Agency for Health and Food Safety, Vienna, Austria; Institute for Surveillance and Infectious Disease Epidemiology, Austrian Agency for Health and Food Safety, Vienna, Austria; Sloan School of Management, Massachusetts Institute of Technology, Cambridge, Massachusetts, USA; Department of Clinical Research, University of Southern Denmark, Odense, Denmark; Departments of Medicine, Epidemiology and Population Health, Biomedical Data Science, and Statistics and Meta-Research Innovation Center at Stanford (METRICS), Stanford University, Stanford, California, USA; Division of Endocrinology and Diabetology, Department of Internal Medicine, Medical University of Graz, Graz, Austria

**Keywords:** COVID-19, mortality, observational study, SARS-CoV-2, vaccine

## Abstract

**Background:**

We aimed to evaluate the effectiveness of severe acute respiratory syndrome coronavirus 2 (SARS-CoV-2) vaccinations in previously SARS-CoV-2–infected adults in the general population of Austria during the Delta wave and with extended follow-up.

**Methods:**

In a nationwide retrospective cohort study, we calculated age-, sex-, and nursing home residency–adjusted Cox proportional hazard ratios (HRs) of coronavirus disease 2019 (COVID-19) deaths, SARS-CoV-2 infections, and non-COVID-19 deaths from 1 October to 31 December 2021, and secondarily with extended follow-up to 30 June 2022. Relative vaccine effectiveness (rVE) is rVE = (1 – HR) × 100.

**Results:**

Among 494 646 previously infected adults, 169 543 had received 2 vaccine doses, 133 567 had received 1 dose, and 190 275 were unvaccinated at baseline. We recorded 17 COVID-19 deaths (6 vaccinated, 11 unvaccinated) and 8209 SARS-CoV-2 infections. Absolute risk of COVID-19 deaths was 0.003%. rVE estimates for COVID-19 deaths and reinfections exceeded 75% until the end of 2021 but decreased substantially with extended follow-up. The risk of non-COVID-19 death was lower in those vaccinated versus unvaccinated.

**Conclusions:**

First and second SARS-CoV-2 vaccine doses appear effective in the short-term, but with diminishing effectiveness over time. The extremely low COVID-19 mortality, regardless of vaccination, indicates strong protection of previous infection against COVID-19 death. Lower non-COVID-19 mortality in the vaccinated population might suggest a healthy vaccinee bias.

Vaccinations against severe acute respiratory syndrome coronavirus 2 (SARS-CoV-2) were critical to mitigate adverse health consequences of the coronavirus disease 2019 (COVID-19) pandemic [1]. Recommendations for primary vaccination against SARS-CoV-2 were based on randomized controlled trials (RCTs) documenting high vaccine efficacy against (symptomatic) SARS-CoV-2 infections in previously uninfected individuals [2]. Despite the dearth of RCT data on vaccine efficacy in previously SARS-CoV-2–infected individuals, the general vaccine policy in 2021 in many countries, including Austria, had been to vaccinate the national population against SARS-CoV-2 regardless of previous infection status.

Evidence is still limited on the effectiveness of the first vaccine dose against SARS-CoV-2 in individuals with a previous infection regarding hard clinical outcomes such as COVID-19 deaths. Most epidemiological studies on this issue have been restricted to mostly uninfected populations, did not stratify for prior infection status, and/or did not report on severe outcomes and COVID-19 deaths in particular [3]. Few studies on hybrid immunity, conferred by a combination of a previous SARS-CoV-2 infection and vaccination, have reported significant vaccine effectiveness of the first vaccination against (symptomatic) reinfection and severe COVID-19 while others have not [4, 5]. Previous studies on the effectiveness of the first SARS-CoV-2 vaccination in previously infected individuals either did not specifically report COVID-19 deaths or refrained from calculating risk estimates due to only a few or no outcome events [6–9]. In addition, most existing studies have not considered a potential healthy vaccinee bias—that those who choose to get vaccinated tend to have a better overall health status reflected by lower non-COVID-19 mortality as compared to individuals who remain unvaccinated [10–12].

In this nationwide study in Austria, we aimed to evaluate the effectiveness of the first and second vaccine dose against SARS-CoV-2 in previously infected adults regarding COVID-19 deaths (primary outcome) and SARS-CoV-2–positive test results (secondary outcome), thus comparing hybrid versus natural immunity. In addition, we calculated risk differences in non-COVID-19 mortality in individuals with versus without a first or second vaccine dose, to determine whether the underlying health status of vaccinated and unvaccinated individuals is comparable or if a healthy vaccinee bias is present. The observation period for this investigation was from 1 October until 31 December 2021, coinciding with the SARS-CoV-2 Delta wave, which marked the largest number of COVID-19 deaths after the introduction of vaccines against SARS-CoV-2 in Austria. We also considered extended follow-up data for an additional 6 months (when the Omicron wave spread in Austria), in order to understand the durability of the effects.

## METHODS

### Study Design, Procedures, and Participants

In this retrospective population-based observational cohort study, we used national health data from the Austrian epidemiological reporting system (German: Epidemiologisches Meldesystem [EMS]) provided by the Austrian Agency for Health and Food Safety (German: Österreichische Agentur für Gesundheit und Ernährungssicherheit [AGES]), which recorded data of all individuals with a documented SARS-CoV-2 infection until 30 June 2023 [13]. Individual participant data were not available for persons without previous documentation of infection. Detection of SARS-CoV-2 infections was based on the EMS that only records polymerase chain rection (PCR) tests or, restricted to the end of 2020 until May 2021, antigen tests from accredited diagnostic facilities. Home antigen tests were not recorded in the EMS and are consequently not included in this study. Unique personal identifiers were used to match the EMS data with individual all-cause mortality data provided by Statistics Austria and with individual vaccination data provided by the national COVID-19 vaccine registry [13]. Residency in Austria was verified by checking the Central Registry of Residence in Austria at the date of reporting of the SARS-CoV-2 infection. Postal address at the time of the SARS-CoV-2 infection was used to classify nursing home residency. No sample size calculation was performed prior to initiation of the study. We followed the Strengthening the Reporting of Observational Studies in Epidemiology (STROBE) checklist (Supplementary Table 1).

The study population encompasses all Austrian residents aged 18 years or older who were previously infected with SARS-CoV-2, had their infection recorded in the EMS, and were alive on 1 October 2021. According to the widely used definition that SARS-CoV-2 reinfections require 2 positive tests separated by >90 days (due to potential long-term viral shedding), we excluded all individuals with a positive SARS-CoV-2 test within 90 days before the observation period [14, 15].

From 1 October to 31 December 2021, we recorded COVID-19 deaths, SARS-CoV-2–positive test results (regardless of symptoms), all-cause mortality, date of vaccination, and vaccine product used in the study population. Analyses of case fatality rates (CFRs) during this follow-up period included not only the study population described above but also individuals without a prior infection, allowing for a comparison of CFRs between previously infected and uninfected individuals. Presence of repeated previous documented infection was classified if there were ≥2 positive test results >90 days apart. COVID-19 deaths were classified in persons with a positive SARS-CoV-2 test who subsequently died due to COVID-19 as recorded by the local public health office.

### Statistical Analysis

Categorical data are presented as percentages and continuous data are shown as medians (with 25th to 75th percentile). We calculated Cox proportional hazard ratios (HRs) with 95% confidence intervals (CIs) in groups according to the number of vaccine doses against SARS-CoV-2. We calculated crude, age (untransformed continuous variable in years) adjusted, age and sex (binary variable) adjusted, as well as age, sex, and nursing home residency (binary variable) adjusted HRs [16]. We assumed that it takes 14 days for the first and second SARS-CoV-2 vaccination to become effective [4]. Thus, individuals changed their group allocation regarding vaccination status 14 days after receiving the vaccine dose.

We conducted sensitivity analyses that excluded the 14-day period and individuals who changed group allocation at the day of vaccination [17]. Another sensitivity analysis excluded individuals with multiple (>1) previous infections. Censoring occurred at the end of the observation period, the date of non-COVID-19 death, the date of a third vaccination, or the date of the outcome event, whatever occurred first. Relative vaccine effectiveness (rVE) was calculated as (1 – HR) × 100.

Group comparisons were performed for the entire study population and for subgroups stratified by sex, age, vaccine type (Pfizer [Comirnaty], Moderna [Spikevax], Janssen [Jcovden], or AstraZeneca [Vaxzevria]), year of the last previous infection, and nursing home residency. Additional subgroup analyses were performed in individuals who were first infected and then vaccinated and vice versa, as individuals who are infected following a vaccination against SARS-CoV-2 may be at particularly high risk for further infections and may introduce a bias toward less or even negative rVE, as documented in a previous study on collider bias [18]. To evaluate potential waning immunity after vaccination, we performed analyses, stratified in those who received the first vaccine dose within 45 days, from >45 to 90 days, >90 to 135 days, >135 to 180 days, and >180 days before 1 October 2021. Finally, analyses were performed for non-COVID-19 mortality with group allocation switching on the day of vaccination [11]. Analyses were prespecified and agreed upon among authors before any data were analyzed. The analysis framework is the same as applied previously by our team to examine the effectiveness of booster doses in the nationwide Austrian data among previously infected individuals [10]. This investigation is part of the Severe Acute Respiratory Syndrome Coronavirus 2 Re-Infection Risk and Vaccine Efficacy Study in Austria (SARIVA) that is registered at ClinicalTrials.gov (NCT06162533).

An extended follow-up period from 1 January 2022 until 30 June 2022 was used to evaluate the durability of vaccine effectiveness against the Omicron variant with regard to COVID-19 deaths and SARS-CoV-2 infections, and to further investigate group differences in non-COVID-19 mortality. The same definitions and analytical processes were used as in the main analysis of 1 October to 31 December 2021.

Proportional hazards assumptions were checked by graphically examining Schoenfeld residuals. In the case of violation, we planned to split the observation period. Cox proportional HRs were only calculated for analyses with at least 10 outcome events. Statistical analyses were performed in R software (version 4.2.2) [19].

## RESULTS

### Study Population

**Figure 1.**
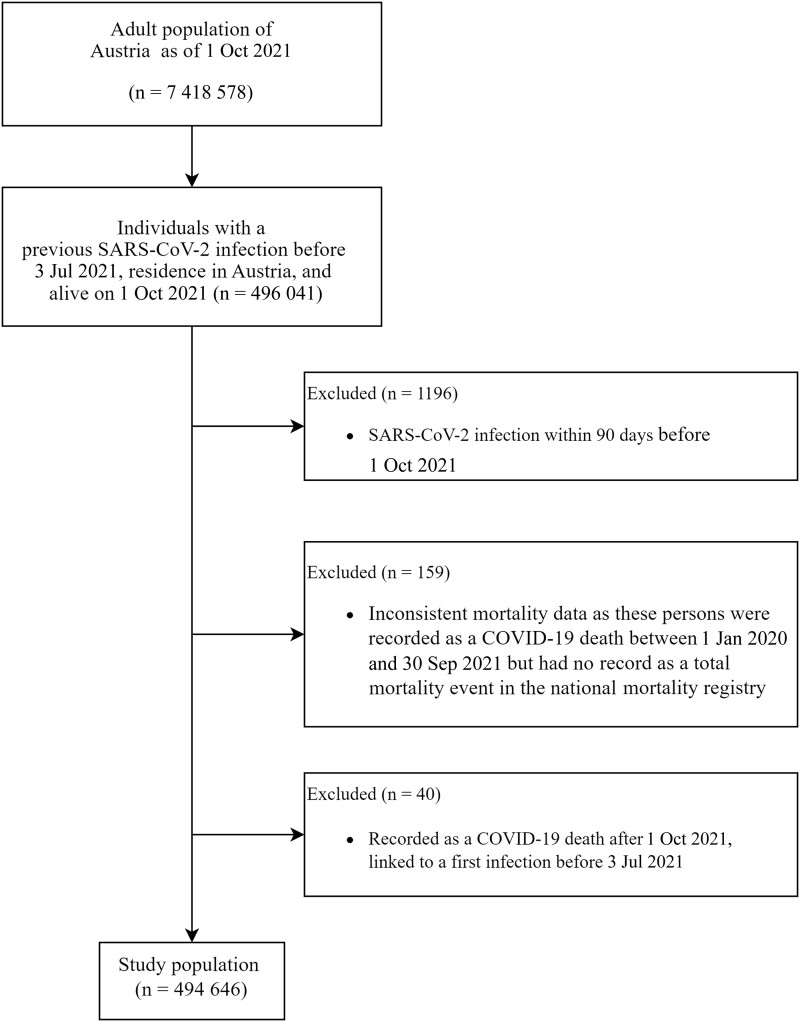
Participant selection chart. Abbreviations: COVID-19, coronavirus disease 2019; SARS-CoV-2, severe acute respiratory syndrome coronavirus 2.

From the adult population in Austria of 7 418 578 (as of 1 October 2021), 494 646 were eligible for study inclusion (Figure 1). Baseline characteristics are presented in Table 1 and Supplementary Table 2. The median age was 45 years, 133 567 had received 1 dose, 169 543 had received 2 doses, and 190 275 were categorized as unvaccinated. Of the study population, 2.5% were nursing home residents. The main differences across groups were that older individuals had received more vaccinations and were vaccinated earlier, with the nursing home population being vaccinated first (Table 1). Time since last vaccination for nursing home residents was on average 227 days while individuals younger than 40 years were on average vaccinated 78 days before the start of the study period (Table 1). Due to the low number of individuals with 3 or more vaccine doses, we refrained from any statistical analyses in this group.

**Table 1.** Baseline Characteristics of the Study Population of Adults in Austria With Previously Documented Severe Acute Respiratory Syndrome Coronavirus 2 Infection as of 1 October 2021

Characteristic	All	Females	Males	Unvaccinated	1 Dose	2 Doses
No. of participants	494 646	255 960	238 686	190 275	133 567	169 543
Female sex	255 960 (51.75%)	255 960 (100.00%)	0 (0.00%)	100 163 (52.64%)	67 787 (50.75%)	87 152 (51.40%)
Age, y	45 (31–57)	45 (32–57)	44 (30–57)	40 (29–53)	46 (32–58)	49 (34–61)
Nursing home residency	12 343 (2.50%)	9295 (3.63%)	3048 (1.28%)	1974 (1.04%)	1638 (1.23%)	7961 (4.70%)
Vaccinations against SARS-CoV-2						
Unvaccinated	190 275 (38.47%)	100 163 (39.13%)	90 112 (37.75%)	190 275 (100.00%)	0 (0.00%)	0 (0.00%)
1 dose	133 567 (27.00%)	67 787 (26.48%)	65 780 (27.56%)	0 (0.00%)	133 567 (100.00%)	0 (0.00%)
2 doses	169 543 (34.28%)	87 152 (34.05%)	82 391 (34.52%)	0 (0.00%)	0 (0.00%)	169 543 (100.00%)
3 or more doses	1261 (0.25%)	858 (0.34%)	403 (0.17%)	0 (0.00%)	0 (0.00%)	0 (0.00%)
Time since last vaccination, d	91 (59–132)	94 (59–140)	88 (58–123)	NA	91 (49–133)	92 (65–132)
Previous SARS-CoV-2 infections						
Single previous infection	493 780 (99.82%)	255 485 (99.81%)	238 295 (99.84%)	189 928 (99.82%)	133 341 (99.83%)	169 258 (99.83%)
Repeated previous infections	866 (0.18%)	475 (0.19%)	391 (0.16%)	347 (0.18%)	226 (0.17%)	285 (0.17%)
Time since last infection, d	283 (201–323)	287 (204–323)	281 (198–323)	247 (184–317)	276 (201–321)	307 (252–328)

Data are presented as No. (%) or median (interquartile range).

Abbreviations: NA, not applicable; SARS-CoV-2, severe acute respiratory syndrome coronavirus 2.

### COVID-19 Deaths

From 1 October to 31 December 2021, we recorded 17 COVID-19 deaths among the previously infected, of which 11 occurred in the unvaccinated group and 6 in the group with 2 vaccine doses. This corresponds to a CFR of 0.21% among 8209 recorded infections, and an absolute risk of COVID-19 death of 0.003% within the study population of 494 646 individuals. In the group with 1 vaccine dose, no COVID-19 death was recorded. COVID-19 deaths after reinfection mainly occurred in the age group ≥75 years (Table 2).

**Table 2.** Number of Coronavirus Disease 2019 Deaths, Severe Acute Respiratory Syndrome Coronavirus 2 (SARS-CoV-2) Infections, and Case Fatality Rate by Presence of Previous Documented SARS-CoV-2 Infection, in the Total Study Population and Subgroups of Age and Nursing Home Residency, 1 October 2021 to 31 December 2021

Characteristic	Previous Infection	No. of COVID-19 Deaths, by Vaccination Status			No. of SARS-CoV-2 Infections, by Vaccination Status			CFR, %, by Vaccination Status		
		0 Doses	1 Dose	2 Doses	0 Doses	1 Dose	2 Doses	0 Doses	1 Dose	2 Doses
Total	No	1619	114	1058	202 417	22 676	158 295	0.80	0.50	0.67
	Yes	11	0	6	6086	796	1327	0.18	0.00	0.45
Nursing home resident	No	191	31	184	572	137	1011	33.39	22.63	18.20
	Yes	3	0	3	40	11	42	7.50	0.00	7.14
Community dwelling	No	1428	83	874	201 845	22 539	157 284	0.71	0.37	0.56
	Yes	8	0	3	6046	785	1285	0.13	0.00	0.23
Age 18–39 y	No	15	0	1	101 695	12 663	53 247	0.01	0.00	0.00
	Yes	1	0	0	3581	428	632	0.03	0.00	0.00
Age 40–59 y	No	135	8	37	78 404	8020	61 870	0.17	0.10	0.06
	Yes	2	0	1	2196	302	496	0.09	0.00	0.20
Age 60–74 y	No	425	31	229	17 163	1422	29 932	2.48	2.18	0.77
	Yes	2	0	0	232	34	111	0.86	0.00	0.00
Age ≥75 y	No	1044	75	791	5155	571	13 246	20.25	13.13	5.97
	Yes	6	0	5	77	32	88	7.79	0.00	5.68

Abbreviations: CFR, case fatality rate; COVID-19, coronavirus disease 2019; SARS-CoV-2, severe acute respiratory syndrome coronavirus 2.

During the same time frame, 2892 COVID-19 deaths were recorded in the remainder of the adult population of Austria with no documented prior SARS-CoV-2 infection (estimated to be 6 758 312 people, by using national population statistics provided by Statistics Austria in combination with EMS data). The absolute risk of COVID-19 deaths was therefore 0.043% compared to 0.003% in our population with previously documented infection. Age-specific CFRs according to vaccination status and by presence or absence of a documented previous infection are depicted in Table 2.

Due to the very low number of COVID-19 deaths in people with previously documented infection, HR estimates should be seen with extra caution. In exploratory calculations, the crude and adjusted (for age, sex, and nursing home residency) HRs for COVID-19 deaths in individuals with 2 doses were 0.42 (95% CI, .15–1.14) and 0.24 (95% CI, .09–.65), respectively, compared with no vaccination. The crude and adjusted HR for COVID-19 deaths in individuals with 1 or 2 doses was 0.23 (95% CI, .09–.63) and 0.16 (95% CI, .06–.43), respectively, compared with no vaccination (Table 3, Supplementary Figure 1).

**Table 3.** Cox Proportional Hazard Ratios With 95% Confidence Intervals for Coronavirus Disease 2019 Deaths and Severe Acute Respiratory Syndrome Coronavirus 2 Infections According to Vaccination Status, 1 October 2021 to 31 December 2021

Characteristic	Unvaccinated	2 Vaccine Doses	1 or 2 Vaccine Doses
COVID-19 deaths, No.	11	6	6
Events per 100 000 person-days	0.08	0.03	0.02
Crude HR (95% CI)	Reference	0.42 (.15–1.14)	0.23 (.09–.63)
Age-adjusted HR (95% CI)	Reference	0.27 (.10–.73)	0.17 (.06–.47)
Age- and sex-adjusted HR (95% CI)	Reference	0.25 (.09–.69)	0.16 (.06–.44)
Age-, sex-, and nursing home residency–adjusted HR (95% CI)	Reference	0.24 (.09–.65)	0.16 (.06–.43)

	Unvaccinated	1 Vaccine Dose	2 Vaccine Doses
SARS-CoV-2 infections, No.	6086	796	1327
Events per 100 000 person-days	46.63	4.52	5.92
Crude HR (95% CI)	Reference	0.12 (.11–.13)	0.16 (.15–.17)
Age-adjusted HR (95% CI)	Reference	0.12 (.11–.13)	0.17 (.16–.19)
Age- and sex-adjusted HR (95% CI)	Reference	0.12 (.11–.13)	0.17 (.16–.19)
Age-, sex-, and nursing home residency–adjusted HR (95% CI)	Reference	0.12 (.12–.13)	0.17 (.16–.18)

Abbreviations: CI, confidence interval; COVID-19, coronavirus disease 2019; HR, hazard ratio; SARS-CoV-2, severe acute respiratory syndrome coronavirus 2.

### SARS-CoV-2 Infections

From 1 October to 31 December 2021, 8209 SARS-CoV-2 infections were recorded in the study population. Median follow-up time for SARS-CoV-2 infections in the unvaccinated, the 1-vaccine dose group, and the 2-vaccine dose group was 84 days, 61 days, and 69 days, respectively.

We observed a significant rVE with 88% (95% CI, 87%–88%) for 1 versus no vaccine dose as well as a significant rVE of 83% (95% CI, 82%–84%) for individuals with 2 vaccine doses when compared to the unvaccinated group (Table 3, Supplementary Figure 1). Subgroup analysis on SARS-CoV-2 infections was restricted to groups with at least 10 outcome events and confirmed a statistically significant rVE for all analyzed subgroups and in all sensitivity analyses. We observed the lowest age-, sex-, and nursing home residency–adjusted rVE in the age group ≥75 years with 63% (95% CI, 45%–76%) for individuals with 1 vaccine dose and 56% (95% CI, 40%–67%) for the group with 2 vaccine doses (Supplementary Table 3). The age-, sex-, and nursing home residency–adjusted rVE of mRNA vaccines (Pfizer [Comirnaty] and Moderna [Spikevax]) were comparable (Supplementary Table 4). The rVE for the AstraZeneca vaccine (Vaxzevria) was lower with 63% (95% CI, 65%–79%) for 1 vaccine dose and 65% (95% CI, 61%–70%) for 2 vaccine doses (Supplementary Table 4). Subgroup analyses on the order of events (infection prior to vaccination and vice versa) showed no significant difference (Supplementary Table 5). SARS-CoV-2 infection rate and rVE was higher in those with a last previous infection in 2020 versus 2021 (Supplementary Table 6).

For SARS-CoV-2 infections, rVE declined with time elapsed since the last vaccination. Initially, rVE was 90% (95% CI, 89%–92%) within the first 45 days of receiving the first vaccination and decreased to 72% (95% CI, 64%–79%) 180 days or more after the first vaccine dose. Similarly, rVE decreased from 90% (95% CI, 88%–92%) within 45 days of the second vaccination to 59% (95% CI, 52%–66%) after 180 days or more since receiving the second vaccine dose (Supplementary Table 7).

### Non-COVID-19 Mortality

In participants with 2, 1, and no vaccinations, we recorded 266, 505, and 350 non-COVID-19–related deaths from 1 October to 31 December 2021, respectively. Compared to unvaccinated individuals, the age-, sex-, and nursing home residency–adjusted HR for non-COVID-19 mortality in those with 1 and 2 vaccinations was 0.73 (95% CI, .62–.85) and 0.64 (95% CI, .56–.74), respectively (Table 4, Supplementary Figure 1).

**Table 4.** Cox Proportional Hazard Ratios With 95% Confidence Intervals for Non-COVID-19 Deaths According to Vaccination Status, 1 October 2021 to 31 December 2021

Characteristic	Unvaccinated	1 Vaccine Dose	2 Vaccine Doses
Deaths (non-COVID-19), No.	350	266	505
Events per 100 000 person-days	2.92	1.6	2.24
Crude HR (95% CI)	Reference	0.72 (.61–.84)	1.07 (.93–1.23)
Age-adjusted HR (95% CI)	Reference	0.70 (.60–.82)	0.70 (.61–.80)
Age and sex-adjusted HR (95% CI)	Reference	0.69 (.59–.81)	0.69 (.60–.79)
Age-, sex-, and nursing home residency–adjusted HR (95% CI)	Reference	0.73 (.62–.85)	0.64 (.56–.74)

Abbreviations: CI, confidence interval; COVID-19, coronavirus disease 2019; HR, hazard ratio.

### Extended Follow-up

On 1 January 2022, which marked about the beginning of the first Omicron wave, our study population with up to 2 vaccine doses was reduced to 392 896 individuals due to third vaccinations, SARS-CoV-2 infections, and mortality from 1 October until 31 December 2021. Group sizes with zero, 1, and 2 vaccine doses were 89 013, 123 964, and 179 919 individuals, respectively. From 1 January 2022 until 30 June 2022, we recorded 46 COVID-19 deaths (28 among vaccinated, 18 among nonvaccinated), 110 762 SARS-CoV-2 infections, and 1286 non-COVID-19 deaths. For COVID-19 mortality and SARS-CoV-2 infections, the rVE was reduced substantially (HRs roughly doubled) when compared against the observation period in 2021 (Supplementary Table 8). Differences in non-COVID-19 mortality between vaccinated versus unvaccinated individuals were reduced to a similar extent when compared to the data from 2021 (Supplementary Table 8).

## DISCUSSION

The Delta variant–dominated SARS-CoV-2 wave in the winter of 2021 was marked by a high number of deaths attributed to COVID-19 in the general population of Austria, but there was an extremely low number of COVID-19 deaths in people with previously documented infection, regardless of vaccination status. We observed significant rVE of the first and second vaccine dose against SARS-CoV-2 reinfections, but rVE declined with time elapsed after the last vaccination. Non-COVID-19 mortality was lower in vaccinated versus unvaccinated individuals, suggesting healthy vaccinee bias. During extended follow-up in the first half-year of 2022, when Omicron was predominant, rVE and group differences in non-COVID-19 mortality were less significant, but overall COVID-19 mortality rate remained at a similarly low level when compared to 2021.

The low number of COVID-19 deaths in our study population throughout the Delta and Omicron waves might indicate a significant protection against this outcome in previously infected individuals. However, this may also be partially due to survivorship bias, as the most fragile individuals may have died from their first infection. Due to limited access to individual participant data of individuals without a previously documented infection, we were not able to perform formal statistical comparisons of previously infected versus uninfected adults. Data on absolute COVID-19 mortality and the CFR are nevertheless consistent with a strong protection of natural immunity, conferred by a previous SARS-CoV-2 infection, against COVID-19 mortality. This notion is in line with some other investigations on this topic and may indicate a relatively long-term protection against COVID-19 mortality, when considering that we excluded all individuals with a previous SARS-CoV-2 infection within 90 days before the study period [20]. Analysis on rVE regarding COVID-19 mortality suggests a significant rVE against COVID-19 deaths, even in previously SARS-CoV-2–infected individuals. However, in view of the very low absolute COVID-19 mortality, one may question the overall clinical significance and the cost-effectiveness of these vaccinations for prevention of COVID-19 deaths [21]. Due to very sparse outcome events, we refrained from calculating numbers needed to vaccinate and cost-effectiveness analyses of these vaccinations, but strongly recommend further studies to address these issues. It should be noted that our analyses do not consider potential vaccination harms or other potential negative health consequences following SARS-CoV-2 infection, except of COVID-19 deaths [22, 23]. Our results are also observational in nature and subject to biases such as healthy vaccinee bias, which our results suggest is present. This would likely overestimate any true vaccine effectiveness.

Our results support previous findings that, even in previously SARS-CoV-2–infected adults, vaccination is associated with a reduction in documented reinfections with waning but still significant effectiveness against the Delta variant >180 days after the last vaccination [6, 7, 24–26]. The vast majority of our study population was vaccinated with the Pfizer vaccine (Comirnaty), which yielded similarly high rVE as the Moderna (Spikevax) vaccine, whereas rVE was lower for the Janssen (Jcovden) vaccine, and lowest for the AstraZeneca (Vaxzevria) vaccine, consistent with other investigations [3]. In subgroup analyses, we observed a lower rVE for individuals aged 75 years or older who are unfortunately at highest risk from COVID-19.

During the Omicron wave in 2022, the rVEs for all outcomes were less strong as compared to 2021, but some effectiveness remained. Underlying reasons for this diminished rVE are speculative but may be due to differences in virus variants, waning, changing population characteristics from 2021 to 2022, or other factors.

In individuals with 1 and 2 vaccine doses, we observed a 27% and 36% lower non-COVID-19 mortality risk, respectively, when compared to unvaccinated individuals. Similarly to rVE, these group differences in non-COVID-19 mortality were less significant in 2022 versus 2021. In general, these data suggest healthy vaccinee bias comparable with other analyses of vaccinated populations [10–12, 27]. This suggests that the vaccinated persons in our study may have been fundamentally different from the unvaccinated and likely had better underlying health. This would have tended to bias our results in the direction of overestimating vaccine effectiveness and highlights the potential challenges of using retrospective observational data to draw causal conclusions about interventions. Mechanistically, our findings on the effectiveness of natural and hybrid immunity are likely mediated by a broad and durable humoral and cellular immunity [28–30]. Long-term protection against severe COVID-19 may be particularly attributed to cellular immunity and less to neutralizing antibodies [28–30].

Our findings are subject to several limitations inherent to observational studies of COVID-19 vaccine effectiveness. Because the vaccination rollout prioritized vulnerable and elderly populations, they were more likely as a group to have received vaccinations earlier. Additionally, there were varying timelines of authorization for different vaccine types in the European Union. Mandatory notification data, which we used, are subject to potential bias and/or confounding. These include limitations in data reporting, misclassification between COVID-19 and non-COVID-19 deaths, and variation on access to and indication for SARS-CoV-2 tests. Although Austria implemented an aggressive testing policy, making PCR tests readily available to all residents, the introduction of home antigen tests—which were not included in official data—could introduce bias. This is because different groups may have used home antigen tests and PCR tests at varying rates. Data on testing frequencies as well as migration data on individuals included in the study are missing. Missing data on negative test results precluded the use of a test-negative study design. Potential behavior changes in response to vaccination and/or SARS-CoV-2 infection, as well as test results, may introduce further biases. Furthermore, we did not have access to data on comorbidities, socioeconomic status, and medications, preventing adjustment for these factors. The fact that we found 1 dose of vaccination to be associated with more protection than 2 alone suggests residual bias. Nevertheless, we utilized national data on nursing home residency, which represents a proxy for many comorbidities and the strongest factor affecting the infection fatality rate. We consider it a main strength of our work that findings significantly add to the very rare nationwide investigations in individuals with hybrid and natural immunity on vaccine effectiveness [5, 7, 26, 31].

Even during the Delta wave, which saw the largest number of deaths attributed to COVID-19 in Austria after the introduction of vaccines, the risk of COVID-19 deaths in the previously infected population was extremely low, and this was sustained even during the first half-year of 2022, when Omicron was predominant. In conclusion, we observed that vaccination was associated with a significant reduction of SARS-CoV-2 infections against the background of very low absolute COVID-19 mortality risks. Further studies on various SARS-CoV-2 infection outcomes are warranted to provide estimates on the public health significance and cost-effectiveness of SARS-CoV-2 vaccines in previously infected individuals.

## Supplementary Material

ofae547_Supplementary_Data

## Supplementary Data

Supplementary materials are available at *Open Forum Infectious Diseases* online. Consisting of data provided by the authors to benefit the reader, the posted materials are not copyedited and are the sole responsibility of the authors, so questions or comments should be addressed to the corresponding author.

## Notes

***Acknowledgments*.** The authors thank all persons and organizations involved in data collection.

***Author contributions*.** A. C. and S. P. conceptualized the study. J. P. A. I. made contributions to the analytical plan. A. C. and L. R. were involved in data curation. A. C. performed the formal analyses. A. C. and S. P. wrote the original draft of the manuscript. All authors contributed to supervision, writing, reviewing, and editing the manuscript, and approved the final version before submission.

***Data availability*.** The data that support the findings of this study are available upon request with approval needed from the Austrian Agency for Health and Food Safety, Vienna, Austria. The data are not publicly available due to restrictions pertaining to contained information that could compromise the privacy of patients.

***Patient consent*.** Ethical approval for this study was obtained from the ethics committee at the Medical University of Graz, Austria (no. 33-144 ex 20/21). No written informed consent from the participants was required.

***Financial support*.** This work was funded by the Austrian Science Fund (grant number KLI 1188).
