# Supplementary material for: Effectiveness of the First and Second Severe Acute Respiratory Syndrome Coronavirus 2 Vaccine Dose: A Nationwide Cohort Study From Austria on Hybrid Versus Natural Immunity
Source: Open Forum Infect Dis. 2024 Sep 19;11(10):ofae547. doi: 10.1093/ofid/ofae547 (PMC11450622; doi:10.1093/ofid/ofae547)

[**Table S1: STROBE Statement—Checklist of items for *cohort studies*** 2](#_Toc175675849)

[**Table S2: Baseline characteristics of the study population of adults in Austria with previously documented infection as of October 1st, 2021** 4](#_Toc175675850)

[**Table S3: Cox proportional hazard ratios (HR) with 95% confidence intervals (95% CI) for SARS-CoV-2 infections from October 1^st^ to December 31^st^, in subgroups according to age and gender** 5](#_Toc175675851)

[**Table S4: Cox proportional hazard ratios (HR) with 95% confidence intervals (95% CI) for SARS-CoV-2 infections from October 1^st^ to December 31^st^, in subgroups according to vaccine product of most recent vaccination** 6](#_Toc175675852)

[**Table S5: Cox proportional hazard ratios (HR) with 95% confidence intervals (95% CI) for SARS-CoV-2 infections according to vaccination status from October 1^st^ to December 31^st^, 2021, according to which immunity conferring event occurred first** 7](#_Toc175675853)

[**Table S6: Cox proportional hazard ratios (HR) with 95% confidence intervals (95% CI) for SARS-CoV-2 infections from October 1^st^ to December 31^st^, in subgroups according to year of the last documented previous SARS-CoV-2 infection** 8](#_Toc175675854)

[**Table S7: Cox proportional hazard ratios (HR) with 95% confidence intervals (95% CI) for SARS-CoV-2 infections from October 1^st^ to December 31^st^, in subgroups according to time since vaccination** 9](#_Toc175675855)

[**Table S8: Cox proportional hazard ratios (HR) with 95% confidence intervals (95% CI) for COVID-19 deaths, SARS-CoV-2 infections, and non-COVID-19 deaths according to vaccination status from January 1^st^ to June 30^st^, 2022** 10](#_Toc175675856)

[**Figure S1: Age, gender and nursing home residency adjusted hazard ratios (95% confidence intervals) for COVID-19 deaths, SARS-CoV-2 infections, and non-COVID-19 deaths according to vaccination status from October 1, 2021, to December 31, 2021** 11](#_Toc175675857)

# **Table S1: STROBE Statement—Checklist of items for *cohort studies***

|  | **Item No** | **Recommendation** | **Main text page** |  |  |
| --- | --- | --- | --- | --- | --- |
| **Title and abstract** | 1 | (*a*) Indicate the study’s design with a commonly used term in the title or the abstract | Page 2, Abstract |  |  |
|  |  | (*b*) Provide in the abstract an informative and balanced summary of what was done and what was found | Page 2, Abstract |  |  |
| **Introduction** | | |  |  |  |
| Background/rationale | 2 | Explain the scientific background and rationale for the investigation being reported | Page 3 and 4, Introduction |  |  |
| Objectives | 3 | State specific objectives, including any prespecified hypotheses | Page 3 and 4, Introduction |  |  |
| **Methods** | | |  |  |  |
| Study design | 4 | Present key elements of study design early in the paper | Page 4 and 5, Methods |  |  |
| Setting | 5 | Describe the setting, locations, and relevant dates, including periods of recruitment, exposure, follow-up, and data collection | Page 4 and 5, Methods |  |  |
| Participants | 6 | (*a*) Give the eligibility criteria, and the sources and methods of selection of participants. Describe methods of follow-up | Page 4 and 5, Methods |  |  |
|  |  | (*b*) For matched studies, give matching criteria and number of exposed and unexposed | Not applicable |  |  |
| Variables | 7 | Clearly define all outcomes, exposures, predictors, potential confounders, and effect modifiers. Give diagnostic criteria, if applicable | Page 4 to 6, Introduction and Methods |  |  |
| Data sources/ measurement | 8* | For each variable of interest, give sources of data and details of methods of assessment (measurement). Describe comparability of assessment methods if there is more than one group | Page 4 and 5, Methods |  |  |
| Bias | 9 | Describe any efforts to address potential sources of bias | Page 5 and 6, national cohort, adjusted statistical analyses |  |  |
| Study size | 10 | Explain how the study size was arrived at | National cohort, no sample size calculation |  |  |
| Quantitative variables | 11 | Explain how quantitative variables were handled in the analyses. If applicable, describe which groupings were chosen and why | Page 5, Methods/Statistical Analysis |  |  |
| Statistical methods | 12 | (*a*) Describe all statistical methods, including those used to control for confounding | Page 5 and 6, Methods/Statistical Analysis |  |  |
|  |  | (*b*) Describe any methods used to examine subgroups and interactions | Page 5 and 6, Methods/Statistical Analysis |  |  |
|  |  | (*c*) Explain how missing data were addressed | no missing data |  |  |
|  |  | (*d*) If applicable, explain how loss to follow-up was addressed | Not applicable |  |  |
|  |  | (*e*) Describe any sensitivity analyses | Page 5 and 6, Methods, Statistical Analysis |  |  |
| **Results** | | |  |  |  |
| Participants | 13* | (a) Report numbers of individuals at each stage of study—eg numbers potentially eligible, examined for eligibility, confirmed eligible, included in the study, completing follow-up, and analysed | Page 7, Results; Table 1 |  |  |
|  |  | (b) Give reasons for non-participation at each stage | Page 4, Methods and Page 8 and 7, Results |  |  |
|  |  | (c) Consider use of a flow diagram | Supplements Figure 1 |  |  |
| Descriptive data | 14* | (a) Give characteristics of study participants (eg demographic, clinical, social) and information on exposures and potential confounders | Table 1, Supplements Table 2 |  |  |
|  |  | (b) Indicate number of participants with missing data for each variable of interest | no missing data |  |  |
|  |  | (c) Summarise follow-up time (eg, average and total amount) | Page 8, Results |  |  |
| Outcome data | 15* | Report numbers of outcome events or summary measures over time | Page 7 and 10, Results; Tables 2 to 4 |  |  |
| Main results | 16 | (*a*) Give unadjusted estimates and, if applicable, confounder-adjusted estimates and their precision (eg, 95% confidence interval). Make clear which confounders were adjusted for and why they were included | Page 7 to 10, Results; Tables 2 to 4 |  |  |
|  |  | (*b*) Report category boundaries when continuous variables were categorized | Page 7 to 10, Results; Tables 2 to 4 |  |  |
|  |  | (*c*) If relevant, consider translating estimates of relative risk into absolute risk for a meaningful time period | Page 8, Results |  |  |
| Other analyses | 17 | Report other analyses done—eg analyses of subgroups and interactions, and sensitivity analyses | Page 7 to 10, Results; Table 5, Supplements Tables 2 to 8 |  |  |
| **Discussion** | | |  |  |  |
| Key results | 18 | Summarise key results with reference to study objectives | Page 10, Discussion |  |  |
| Limitations | 19 | Discuss limitations of the study, taking into account sources of potential bias or imprecision. Discuss both direction and magnitude of any potential bias | Page 12 to 13, Discussion |  |  |
| Interpretation | 20 | Give a cautious overall interpretation of results considering objectives, limitations, multiplicity of analyses, results from similar studies, and other relevant evidence | Page 13, Discussion |  |  |
| Generalisability | 21 | Discuss the generalisability (external validity) of the study results | This is a nationwide survey |  |  |
| **Other information** | | |  |  |  |
| Funding | 22 | Give the source of funding and the role of the funders for the present study and, if applicable, for the original study on which the present article is based | Page 14 |  |  |

*Give information separately for exposed and unexposed groups.

**Note:** An Explanation and Elaboration article discusses each checklist item and gives methodological background and published examples of transparent reporting. The STROBE checklist is best used in conjunction with this article (freely available on the Web sites of PLoS Medicine at http://www.plosmedicine.org/, Annals of Internal Medicine at http://www.annals.org/, and Epidemiology at http://www.epidem.com/). Information on the STROBE Initiative is available at <http://www.strobe-statement.org>.

| **Table S2: Baseline characteristics of the study population of adults in Austria with previously documented infection as of October 1st, 2021** | | | | |
| --- | --- | --- | --- | --- |
|  | **Nursing home residency** | **≥ 75 years** | **60 to < 75 years** | **40 to < 60 years** |
| Number | 12,343 | 37,447 | 64,209 | 191,299 |
| Females (%) | 9,295 (75.31%) | 23,696 (63.28%) | 31,311 (48.76%) | 99,828 (52.18%) |
| Age (years) | 85 (79 - 90) | 82 (78 - 87) | 65 (62 - 69) | 50 (45 - 55) |
| Nursing home residency (%) | 12,343 (100.00%) | 10,599 (28.30%) | 1,744 (2.72%) | 0 (0.00%) |
| Vaccinations against SARS-CoV-2: |  |  |  |  |
| Unvaccinated (%) | 1,974 (15.99%) | 8,131 (21.71%) | 18,103 (28.19%) | 70,788 (37.00%) |
| One vaccine dose (%) | 1,638 (13.27%) | 9,445 (25.22%) | 19,473 (30.33%) | 54,234 (28.35%) |
| Two vaccine doses (%) | 7,961 (64.50%) | 19,096 (50.99%) | 26,444 (41.18%) | 66,077 (34.54%) |
| Three or more vaccine doses (%) | 770 (6.24%) | 775 (2.07%) | 189 (0.29%) | 200 (0.10%) |
| Time since last vaccination (days) | 227 (167 - 238) | 163 (91 - 206) | 105 (66 - 139) | 92 (61 - 127) |
| Single previous infection (%) | 12,272 (99.42%) | 37,338 (99.71%) | 64,112 (99.85%) | 191,018 (99.85%) |
| Repeated previous infections (%) | 71 (0.58%) | 109 (0.29%) | 97 (0.15%) | 281 (0.15%) |
| Time since last infection (days) | 300 (269 - 318) | 294 (240 - 320) | 285 (205 - 322) | 284 (201 - 323) |
|  | **Single previous infection** | **Repeated previous infection** | **Most recent prior infection in 2020** | **Most recent prior infection in 2021** |
| Number | 493,780 | 866 | 265,568 | 229,078 |
| Females (%) | 255,485 (51.74%) | 475 (54.85%) | 140,635 (52.96%) | 115,325 (50.34%) |
| Age (years) | 45 (31 - 57) | 43 (28 - 58) | 46 (31 - 58) | 44 (31 - 56) |
| Nursing home residency (%) | 12,272 (2.49%) | 71 (8.20%) | 8,959 (3.37%) | 3,384 (1.48%) |
| Vaccinations against SARS-CoV-2: |  |  |  |  |
| Unvaccinated (%) | 189,928 (38.46%) | 347 (40.07%) | 81,880 (30.83%) | 108,395 (47.32%) |
| One vaccine dose (%) | 133,341 (27.00%) | 226 (26.10%) | 68,026 (25.62%) | 65,541 (28.61%) |
| Two vaccine doses (%) | 169,258 (34.28%) | 285 (32.91%) | 114,723 (43.20%) | 54,820 (23.93%) |
| Three or more vaccine doses (%) | 1,253 (0.25%) | 8 (0.92%) | 939 (0.35%) | 322 (0.14%) |
| Time since last vaccination (days) | 91 (59 - 132) | 93 (55 - 147) | 105 (73 - 142) | 69 (42 - 106) |
| Single previous infection (%) | 493,780 (100.00%) | 0 (0.00%) | 265,535 (99.99%) | 228,245 (99.64%) |
| Repeated previous infections (%) | 0 (0.00%) | 866 (100.00%) | 33 (0.01%) | 833 (0.36%) |
| Time since last infection (days) | 284 (202 - 323) | 177 (149 - 201.75) | 322 (305 - 334) | 197 (173 - 230) |
| **Vaccine product of most recent vaccination** | **Comirnaty** | **Spikevax** | **Jcovden** | **Vaxzevria** |
| Number | 223,548 | 25,296 | 30,914 | 24,613 |
| Females (%) | 116,568 (52.14%) | 11,728 (46.36%) | 17,285 (55.91%) | 10,216 (41.51%) |
| Age (years) | 48 (33 - 60) | 48 (35 - 58) | 48 (33 - 59) | 41 (28 - 54) |
| Nursing home residency (%) | 9,569 (4.28%) | 550 (2.17%) | 106 (0.34%) | 144 (0.59%) |
| Vaccinations against SARS-CoV-2: |  |  |  |  |
| Unvaccinated (%) | 0 (0.00%) | 0 (0.00%) | 0 (0.00%) | 0 (0.00%) |
| One vaccine dose (%) | 89,871 (40.20%) | 10,022 (39.62%) | 9,176 (29.68%) | 24,498 (99.53%) |
| Two vaccine doses (%) | 132,485 (59.26%) | 15,217 (60.16%) | 21,735 (70.31%) | 106 (0.43%) |
| Three or more vaccine doses (%) | 1,192 (0.53%) | 57 (0.23%) | 3 (0.01%) | 9 (0.04%) |
| Time since last vaccination (days) | 89 (57 - 131) | 99 (71 - 141) | 123 (92 - 147) | 68 (41 - 91) |
| Single previous infection (%) | 223,175 (99.83%) | 25,249 (99.81%) | 30,865 (99.84%) | 24,563 (99.80%) |
| Repeated previous infections (%) | 373 (0.17%) | 47 (0.19%) | 49 (0.16%) | 50 (0.20%) |
| Data are n (%) or median (interquartile range). | | | | |

| **Table S3: Cox proportional hazard ratios (HR) with 95% confidence intervals (95% CI) for SARS-CoV-2 infections from October 1^st^ to December 31^st^, in subgroups according to age and gender** | | | | | | |
| --- | --- | --- | --- | --- | --- | --- |
|  | **Unvaccinated** | **One vaccine dose** | **Two vaccine doses** | **Unvaccinated** | **One vaccine dose** | **Two vaccine doses** |
|  | **≥75 years** | | | **60 to <75 years** | | |
| SARS-CoV-2 infections (n) | 77 | 32 | 88 | 232 | 34 | 111 |
| Events per 100.000 person days | 13.56 | 3.52 | 4.75 | 18.59 | 1.6 | 3.24 |
| Crude HR (95% CI) | Reference | 0.33 (0.22 - 0.49) | 0.46 (0.34 - 0.63) | Reference | 0.10 (0.07 - 0.15) | 0.22 (0.18 - 0.28) |
| Age adjusted HR (95% CI) | Reference | 0.34 (0.23 - 0.52) | 0.48 (0.35 - 0.65) | Reference | 0.10 (0.07 - 0.15) | 0.23 (0.18 - 0.29) |
| Age and gender adjusted HR (95% CI) | Reference | 0.34 (0.23 - 0.52) | 0.47 (0.35 - 0.64) | Reference | 0.10 (0.07 - 0.15) | 0.23 (0.18 - 0.28) |
| Age, gender, and nursing home residency adjusted HR (95% CI) | Reference | 0.36 (0.24 - 0.55) | 0.43 (0.32 - 0.59) | Reference | 0.10 (0.07 - 0.15) | 0.23 (0.18 - 0.28) |
|  | **40 to < 60 years** | | | **18 to < 40 years** | | |
| SARS-CoV-2 infections (n) | 2,196 | 302 | 496 | 3,581 | 428 | 632 |
| Events per 100.000 person days | 45.23 | 4.35 | 5.59 | 56.12 | 5.59 | 7.65 |
| Crude HR (95% CI) | Reference | 0.11 (0.10 – 0.13) | 0.16 (0.14 - 0.17) | Reference | 0.13 (0.11 – 0.14) | 0.17 (0.16 - 0.19) |
| Age adjusted HR (95% CI) | Reference | 0.12 (0.10 - 0.13) | 0.16 (0.15 - 0.18) | Reference | 0.13 (0.11 - 0.14) | 0.17 (0.16 - 0.19) |
| Age and gender adjusted HR (95% CI) | Reference | 0.12 (0.10 - 0.13) | 0.16 (0.15 - 0.18) | Reference | 0.13 (0.11 - 0.14) | 0.17 (0.16 - 0.19) |
| Age, gender, and nursing home residency adjusted HR (95% CI) | Reference | 0.12 (0.10 – 0.13) | 0.16 (0.15 - 0.18) | Reference | 0.13 (0.11 – 0.14) | 0.17 (0.16 - 0.19) |
|  | **Males** | | | **Females** | | |
| SARS-CoV-2 infections (n) | 2,839 | 353 | 605 | 3,247 | 443 | 722 |
| Events per 100.000 person days | 46.54 | 4.08 | 5.49 | 46.71 | 4.94 | 6.34 |
| Crude HR (95% CI) | Reference | 0.10 (0.09 – 0.12) | 0.15 (0.13 – 0.16) | Reference | 0.13 (0.12 – 0.15) | 0.17 (0.16 – 0.19) |
| Age adjusted HR (95% CI) | Reference | 0.11 (0.10 - 0.12) | 0.16 (0.15 - 0.18) | Reference | 0.14 (0.12 - 0.15) | 0.19 (0.17 - 0.20) |
| Age and nursing home residency adjusted HR (95% CI) | Reference | 0.11 (0.10 – 0.12) | 0.16 (0.15 – 0.17) | Reference | 0.14 (0.13 – 0.15) | 0.19 (0.17 – 0.20) |

| **Table S4: Cox proportional hazard ratios (HR) with 95% confidence intervals (95% CI) for SARS-CoV-2 infections from October 1^st^ to December 31^st^, in subgroups according to vaccine product of most recent vaccination** | | | | | | |
| --- | --- | --- | --- | --- | --- | --- |
|  | **Unvaccinated** | **One vaccine dose** | **Two vaccine doses** | **Unvaccinated** | **One vaccine dose** | **Two vaccine doses** |
|  | **Vaccine product of most recent vaccine dose** | | | | | |
|  | **Comirnaty** | | | **Spikevac** | | |
| SARS-CoV-2 infections (n) | 6,086 | 521 | 1,004 | 6,086 | 45 | 73 |
| Events per 100.000 person days | 46.63 | 3.77 | 5.3 | 46.63 | 4.35 | 4.07 |
| Crude HR (95% CI) | Reference | 0.10 (0.10 - 0.11) | 0.15 (0.14 - 0.16) | Reference | 0.11 (0.08 - 0.14) | 0.10 (0.08 - 0.13) |
| Age adjusted HR (95% CI) | Reference | 0.11 (0.10 - 0.12) | 0.16 (0.15 - 0.17) | Reference | 0.11 (0.08 - 0.15) | 0.11 (0.09 - 0.14) |
| Age and gender adjusted HR (95% CI) | Reference | 0.11 (0.10 - 0.12) | 0.16 (0.15 - 0.17) | Reference | 0.11 (0.08 - 0.15) | 0.11 (0.09 - 0.14) |
| Age, gender, and nursing home residency adjusted HR (95% CI) | Reference | 0.11 (0.10 - 0.12) | 0.16 (0.15 - 0.17) | Reference | 0.11 (0.08 - 0.15) | 0.11 (0.09 - 0.14) |
|  | **Jcovden** | | | **Vaxzevria** | | |
| SARS-CoV-2 infections (n) | 6,086 | 172 | 4 | 6,086 | 58 | 246 |
| Events per 100.000 person days | 46.63 | 7.75 | 9.59 | 46.63 | 10.64 | 15.24 |
| Crude HR (95% CI) | Reference | 0.16 (0.14 - 0.19) | 0.35 (0.13 - 0.94) | Reference | 0.24 (0.19 - 0.32) | 0.32 (0.28 - 0.36) |
| Age adjusted HR (95% CI) | Reference | 0.16 (0.14 - 0.19) | 0.38 (0.14 - 1.00) | Reference | 0.27 (0.21 - 0.35) | 0.35 (0.30 - 0.39) |
| Age and gender adjusted HR (95% CI) | Reference | 0.16 (0.14 - 0.19) | 0.38 (0.14 - 1.00) | Reference | 0.27 (0.21 - 0.35) | 0.35 (0.30 - 0.39) |
| Age, gender, and nursing home residency adjusted HR (95% CI) | Reference | 0.16 (0.14 - 0.19) | 0.38 (0.14 - 1.01) | Reference | 0.27 (0.21 - 0.35) | 0.35 (0.30 - 0.39) |

| **Table S5: Cox proportional hazard ratios (HR) with 95% confidence intervals (95% CI) for SARS-CoV-2 infections according to vaccination status from October 1^st^ to December 31^st^, 2021, according to which immunity conferring event occurred first** | | | |
| --- | --- | --- | --- |
|  | **Restricted to infection as first immunity conferring event** | | |
|  | **Unvaccinated** | **One vaccine dose** | **Two vaccine doses** |
| SARS-CoV-2 infections (n) | 6,086 | 785 | 1,270 |
| Events per 100.000 person days | 46.63 | 4.48 | 5.83 |
| Crude HR (95% CI) | Reference | 0.12 (0.11 – 0.13) | 0.16 (0.15 – 0.17) |
| Age adjusted HR (95% CI) | Reference | 0.12 (0.11 - 0.13) | 0.17 (0.16 - 0.18) |
| Age and gender adjusted HR (95% CI) | Reference | 0.12 (0.11 - 0.13) | 0.17 (0.16 - 0.18) |
| Age, gender, and nursing home residency adjusted HR (95% CI) | Reference | 0.12 (0.11 – 0.13) | 0.17 (0.16 – 0.18) |
|  | **Restricted to vaccination as first immunity conferring event** | | |
|  | **Unvaccinated** | **One vaccine dose** | **Two vaccine doses** |
| SARS-CoV-2 infections (n) | 6,086 | 206 | 97 |
| Events per 100.000 person days | 46.63 | 2.55 | 3.38 |
| Crude HR (95% CI) | Reference | 0.10 (0.08 - 0.11) | 0.15 (0.12 - 0.19) |
| Age adjusted HR (95% CI) | Reference | 0.10 (0.08 - 0.11) | 0.16 (0.13 - 0.20) |
| Age and gender adjusted HR (95% CI) | Reference | 0.10 (0.08 - 0.11) | 0.16 (0.13 - 0.20) |
| Age, gender, and nursing home residency adjusted HR (95% CI) | Reference | 0.10 (0.08 - 0.11) | 0.16 (0.13 - 0.20) |

| **Table S6: Cox proportional hazard ratios (HR) with 95% confidence intervals (95% CI) for SARS-CoV-2 infections from October 1^st^ to December 31^st^, in subgroups according to year of the last documented previous SARS-CoV-2 infection** | | | | | | |
| --- | --- | --- | --- | --- | --- | --- |
|  | **2020** | | | **2021** | | |
|  | **Unvaccinated** | **One vaccine dose** | **Two vaccine doses** | **Unvaccinated** | **One vaccine dose** | **Two vaccine doses** |
| SARS-CoV-2 infections (n) | 3,765 | 460 | 867 | 2,321 | 336 | 460 |
| Events per 100.000 person days | 63.55 | 5.85 | 6.36 | 32.56 | 3.44 | 5.24 |
| Crude HR (95% CI) | Reference | 0.11 (0.10 - 0.13) | 0.13 (0.12 - 0.14) | Reference | 0.13 (0.11 - 0.14) | 0.21 (0.19 - 0.23) |
| Age adjusted HR (95% CI) | Reference | 0.12 (0.11 - 0.13) | 0.14 (0.13 - 0.15) | Reference | 0.13 (0.12 - 0.15) | 0.22 (0.20 - 0.25) |
| Age and gender adjusted HR (95% CI) | Reference | 0.12 (0.11 - 0.13) | 0.14 (0.13 - 0.15) | Reference | 0.13 (0.12 - 0.15) | 0.22 (0.20 - 0.25) |
| Age, gender, and nursing home residency adjusted HR (95% CI) | Reference | 0.12 (0.11 - 0.13) | 0.14 (0.13 - 0.15) | Reference | 0.13 (0.12 - 0.15) | 0.22 (0.20 - 0.24) |

| **Table S7: Cox proportional hazard ratios (HR) with 95% confidence intervals (95% CI) for SARS-CoV-2 infections from October 1^st^ to December 31^st^, in subgroups according to time since vaccination** | | | | | | |
| --- | --- | --- | --- | --- | --- | --- |
|  | **Unvaccinated** | **within 45 days** | **> 45 to 90 days** | **> 90 to 135 days** | **> 135 to 180 days** | **> 180 days** |
|  | **Time since first vaccine dose before October 1^st^, 2021** | | | | | |
| SARS-CoV-2 infections (n) | 6,085 | 133 | 175 | 189 | 92 | 49 |
| Events per 100.000 person days | 46.85 | 4.42 | 6.33 | 6.62 | 7.02 | 9.87 |
| Crude HR (95% CI) | Reference | 0.09 (0.08 - 0.11) | 0.13 (0.11 - 0.15) | 0.14 (0.12 - 0.16) | 0.15 (0.12 - 0.19) | 0.24 (0.18 - 0.31) |
| Age adjusted HR (95% CI) | Reference | 0.10 (0.08 - 0.11) | 0.13 (0.11 - 0.15) | 0.14 (0.12 - 0.17) | 0.19 (0.15 - 0.23) | 0.28 (0.21 - 0.37) |
| Age and gender adjusted HR (95% CI) | Reference | 0.10 (0.08 - 0.11) | 0.13 (0.11 - 0.15) | 0.14 (0.12 - 0.17) | 0.19 (0.15 - 0.23) | 0.28 (0.21 - 0.37) |
| Age, gender, and nursing home residency adjusted HR (95% CI) | Reference | 0.10 (0.08 - 0.11) | 0.13 (0.11 - 0.15) | 0.14 (0.13 - 0.17) | 0.19 (0.15 - 0.23) | 0.28 (0.21 - 0.36) |
|  | **Time since second vaccine dose before October 1^st^, 2021** | | | | | |
| SARS-CoV-2 infections (n) | 6,085 | 108 | 419 | 395 | 112 | 159 |
| Events per 100.000 person days | 46.85 | 4.96 | 7.62 | 10.52 | 11.36 | 12.86 |
| Crude HR (95% CI) | Reference | 0.10 (0.08 - 0.12) | 0.15 (0.14 - 0.17) | 0.22 (0.19 - 0.24) | 0.25 (0.21 - 0.30) | 0.31 (0.27 - 0.37) |
| Age adjusted HR (95% CI) | Reference | 0.10 (0.08 - 0.12) | 0.15 (0.14 - 0.17) | 0.25 (0.22 - 0.27) | 0.30 (0.25 - 0.36) | 0.42 (0.36 - 0.50) |
| Age and gender adjusted HR (95% CI) | Reference | 0.10 (0.08 - 0.12) | 0.15 (0.14 - 0.17) | 0.25 (0.22 - 0.27) | 0.30 (0.25 - 0.36) | 0.42 (0.36 - 0.50) |
| Age, gender, and nursing home residency adjusted HR (95% CI) | Reference | 0.10 (0.08 - 0.12) | 0.15 (0.14 - 0.17) | 0.25 (0.22 - 0.28) | 0.30 (0.25 - 0.36) | 0.41 (0.34 - 0.48) |

| **Table S8: Cox proportional hazard ratios (HR) with 95% confidence intervals (95% CI) for COVID-19 deaths, SARS-CoV-2 infections, and non-COVID-19 deaths according to vaccination status from January 1^st^ to June 30^st^, 2022** | | | |
| --- | --- | --- | --- |
|  | **Unvaccinated** | **One vaccine dose** | **Two vaccine doses** |
| COVID-19 deaths (n) | 18 | 5 | 23 |
| Events per 100.000 person days | 0.13 | 0.03 | 0.07 |
| Crude HR (95% CI) | Reference | 0.27 (0.10 - 0.72) | 0.58 (0.31 - 1.07) |
| Age adjusted HR (95% CI) | Reference | 0.37 (0.14 - 1.01) | 0.47 (0.25 - 0.87) |
| Age and gender adjusted HR (95% CI) | Reference | 0.36 (0.13 - 0.98) | 0.46 (0.25 - 0.86) |
| Age, gender, and nursing home residency adjusted HR (95% CI) | Reference | 0.41 (0.15 - 1.11) | 0.47 (0.25 - 0.87) |
|  | **Unvaccinated** | **One vaccine dose** | **Two vaccine doses** |
| SARS-CoV-2 infections (n) | 42,767 | 24,593 | 43,402 |
| Events per 100.000 person days | 496.67 | 214.24 | 146.28 |
| Crude HR (95% CI) | Reference | 0.43 (0.42 - 0.43) | 0.35 (0.34 - 0.35) |
| Age adjusted HR (95% CI) | Reference | 0.41 (0.41 - 0.42) | 0.36 (0.35 - 0.36) |
| Age and gender adjusted HR (95% CI) | Reference | 0.42 (0.41 - 0.42) | 0.36 (0.35 - 0.36) |
| Age, gender, and nursing home residency adjusted HR (95% CI) | Reference | 0.42 (0.41 - 0.42) | 0.36 (0.35 - 0.36) |
|  | **Unvaccinated** | **One vaccine dose** | **Two vaccine doses** |
| Deaths (non-COVID-19) (n) | 339 | 191 | 756 |
| Events per 100.000 person days | 2.41 | 1.42 | 2.24 |
| Crude HR (95% CI) | Reference | 0.59 (0.49 - 0.70) | 1.00 (0.88 - 1.14) |
| Age adjusted HR (95% CI) | Reference | 0.82 (0.69 - 0.99) | 0.83 (0.73 - 0.94) |
| Age and gender adjusted HR (95% CI) | Reference | 0.82 (0.68 - 0.98) | 0.82 (0.72 - 0.93) |
| Age, gender, and nursing home residency adjusted HR (95% CI) | Reference | 0.89 (0.74 - 1.06) | 0.83 (0.73 - 0.94) |

# **Figure S1: Age, gender and nursing home residency adjusted hazard ratios (95% confidence intervals) for COVID-19 deaths, SARS-CoV-2 infections, and non-COVID-19 deaths according to vaccination status, with the unvaccinated group as the reference, from October 1, 2021, to December 31, 2021**


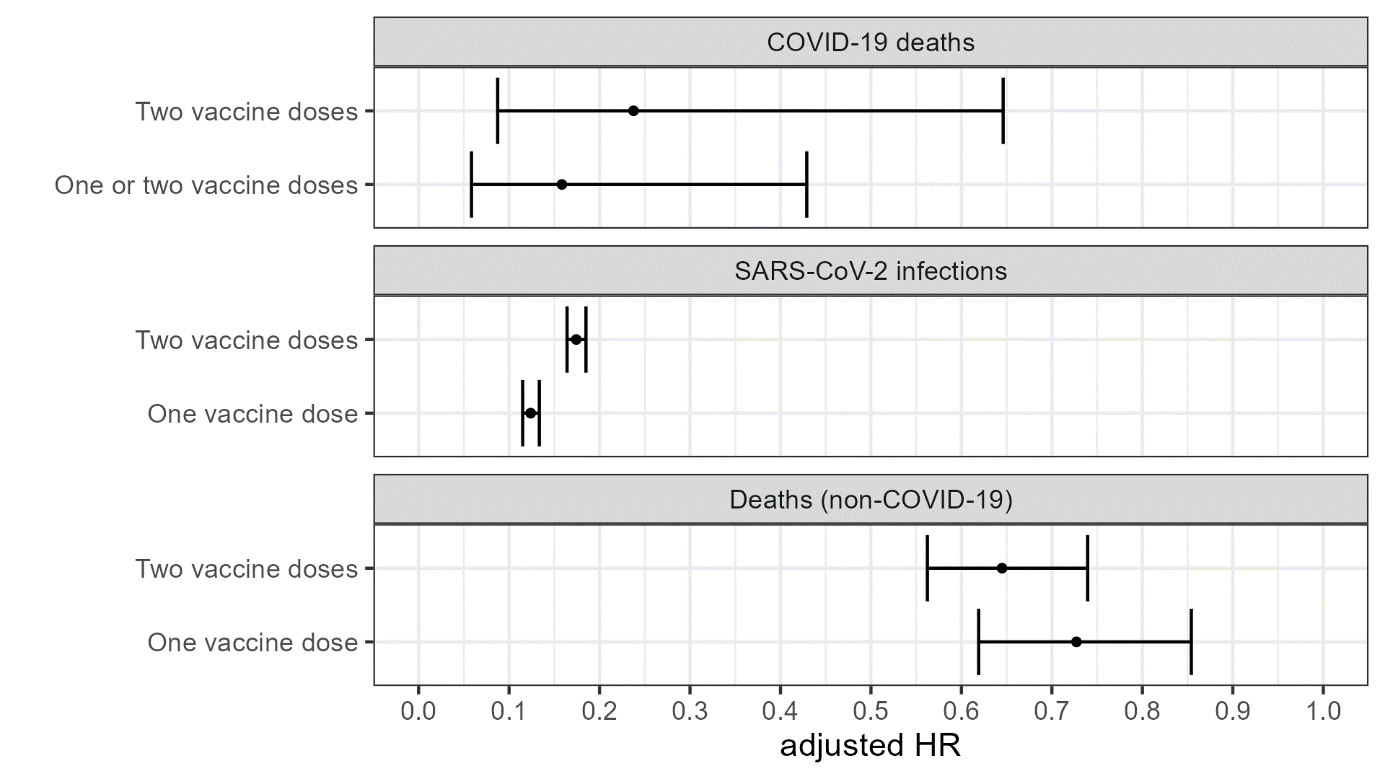

Supplement: ofae547_Supplementary_Data [file ofae547_supplementary_data.docx]
